# Civilian science: the potential of participatory environmental monitoring in areas affected by armed conflicts

**DOI:** 10.1007/s10661-019-7773-9

**Published:** 2019-09-06

**Authors:** Doug Weir, Dan McQuillan, Robert A. Francis

**Affiliations:** 1 The Conflict and Environment Observatory, The Chapel, Scout Road, Hebden Bridge, West Yorkshire HX7 5HZ UK; 2 Department of Computing, Goldsmiths, University of London, New Cross, London, SE14 6NW UK; 3 Department of Geography, King’s College London, Strand Campus, Bush House (North East Wing), 30 Aldwych, London, WC2B 4BG UK

**Keywords:** Citizen science, Warfare, Environment, Data, Monitoring, Assessment, Armed conflict, Peacebuilding

## Abstract

Legal and policy initiatives to address the environmental dimensions of armed conflicts and their impact on people, ecosystems and sustainable development are highly dependent on the availability of environmental data from conflict-affected areas. Socio-political and security conditions in these areas often impede data collection, while traditional models of post-conflict environmental assessments are limited in scope. In response, an increasing range of actors is utilising remote sensing and open source data collection to identify and estimate health and ecological risks during and after conflicts. This paper considers the role of participatory citizen science methodologies in complementing both remote monitoring and post-conflict assessments. It examines existing models and mechanisms for environmental data collection and utilisation in conflict contexts, and the extent to which the core values and principles of citizen science are transferable. We find that ‘civilian science’ is feasible and could be well-suited to conflict conditions. In addition to addressing gaps in data collection, it may also empower communities affected by environmental degradation, enhance their environmental human rights, supplement the often limited monitoring capacity of governmental agencies and facilitate cooperation and peacebuilding. The paper concludes by proposing methodological approaches for three common forms of environmental degradation associated with armed conflicts.

## Introduction

The past decade has witnessed growing interest in the environmental dimensions of armed conflicts among governments, academia, policy-makers, international organisations and civil society (UNEP 2015; UNEA 2016; Daskin and Pringle 2018). This has sought to address the environment throughout the cycle of conflicts: as a risk factor for their onset, as a victim of warfare and as a tool for building peace. Research and policy-making increasingly make little distinction between the humanitarian and environmental consequences of armed conflicts, rightly acknowledging that ecosystems, human health and livelihoods, and sustainable development are inextricably linked (e.g. Baumann and Kuemmerle 2016; Gaynor et al. 2016).

These linkages are particularly apparent when viewed through the lens of human rights, where the role of environmental quality in the enjoyment of fundamental rights, such as those to life or health, is widely accepted—even in situations of armed conflict (UNEA 2016). Underpinning these fundamental rights are procedural rights, for example that environmental information be made public, for public participation in environmental decision-making and to ensure access to effective remedies (UNHRC 2018). Environmental data collection, and public access to that information, is therefore a vital foundation for the protection of human rights.

Environmental data collection, analysis and dissemination can be challenging even in ideal conditions, and in the context of armed conflict especially problematic. There is therefore a need for the development of innovative methods and means to facilitate data collection, so that interventions, whether internally or externally performed, can be based on the best evidence available. In the ecological and environmental sciences more generally, there has been a recent increase in citizen science, wherein public(s) are engaged in a scientific project that produces reliable data for use by scientists, decision-makers or the public (McKinley et al. 2017), and which has substantial potential for addressing complex environmental problems.

This paper explores some of the challenges and constraints in collecting evidence on the environmental impacts of armed conflicts, before examining two innovations that may help to overcome these: the use of remote sensing technologies and, more crucially, whether peacetime approaches to participatory citizen science could inform new field assessment methodologies for data collection on environmental risks and impacts during and after armed conflicts. In doing so, it (1) considers how the socio-political factors that have underpinned the growth in citizen science in peacetime relate to the specific context of armed conflicts; (2) assesses the role of the civilian as citizen, and the means through which participation could be supported by external actors; (3) reviews the constraints and utility of relevant sensors and technologies; and (4) proposes three initial avenues through which ‘civilian science’ could be developed.

## Armed conflicts and the environment: impacts and evidence

Six principal pathways for direct damage to the environment from armed conflicts have been identified, as follows (Jensen and Lonergan 2012): (1) toxic hazards from damage to industrial and urban infrastructure; (2) the legacy of the use of weapons and munitions; (3) human displacement, which can intensify or prolong pressure on ecosystem resources; (4) the use of extractive industries (e.g. logging, mining) to fund conflict; (5) the loss or disruption of water, sanitation and waste infrastructure and (6) the deliberate targeting of natural resources to cause environmental damage. In addition, by disrupting social, economic and administrative systems, conflicts often have indirect effects on the environment. This can include compelling communities to utilise environmentally harmful coping strategies such as artisanal oil refining (Zwijnenburg 2016) or disrupting governmental institutions, undermining the enforcement of laws and initiatives intended to prevent environmental degradation, promote conservation and protect public health (e.g. Dudley et al. 2002; Bruch et al. 2016). In so doing, armed conflict creates and sustains the social and political conditions that degrade systems of environmental governance and stewardship, impeding the environmental dimensions of sustainable development.

These impacts are not new phenomena. However, increasing urbanisation and industrialisation, the growing pressures of population growth, biodiversity loss and climate change, as well as global efforts to achieve sustainable development, are making it increasingly important that the environment is fully addressed throughout the cycle of conflicts. Environmental degradation caused by conflicts can generate acute and chronic risks to human health and ecosystems, can impede post-conflict recovery and reconstruction, and can hamper peacebuilding by sustaining grievances between affected communities and local and national authorities.

In some cases, anticipated health or environmental impacts may be minor, or difficult to determine, and are therefore considered only a low priority for robust evidence collection given the effort required. This may occur where (1) risks are perceived as being low and the nature of evidence collection would be complex, for example depleted uranium exposure, which would involve exposure studies and longitudinal monitoring (McDiarmid et al. 2004); (2) the acute humanitarian impacts of conflict overshadow consideration of other, chronic environmental and health impacts, for example impacts from air pollution in Afghanistan (Weir 2018); (3) impacts and their solutions are systemic or institutional and related to wider socio-political dynamics, so that while conflict may exacerbate the impacts, their complex nature discourages investigation and action; (4) no data are available so risks are not considered, for example exposure to energetic materials or metals from weapons in bombed areas (Savabieasfahani et al. 2016); (5) risk assessment actors promote particular risks to the detriment of others; (6) limited funding for evidence collection and impact remediation is available, so that only sites with highest risk are considered, even though other sites would contain risks that would be unacceptable in peacetime (Pearson 2004); (7) ‘bad actors’ do not want research or evidence collection to take place, and so, data is withheld (Oakford 2017).

Whether prioritised or not, a growing body of research suggests that health and environmental impacts can be pervasive (e.g. Certini et al. 2013), even if the evidence base does need reinforcement. The collection, dissemination and effective utilisation of environmental data is vital to address these problems, but conflicts place a number of constraints on these processes.

### Constraint 1: Politicisation and lack of access for evidence collection

All conflicts operate within the political realm, complicating non-partisan and objective assessment of conflict-related impacts; a process further obfuscated by lack of access due to security risks. Early studies on the environmental legacy of conflicts tended to be driven by the need to map the consequences of specific practices or incidents, for example Cornell University’s study of the legacy of the use of chemical defoliants in the Vietnam War (Littauer and Uphoff 1972; Westing 1975) or Greenpeace’s assessment of the 1991 Gulf War oil fires (Greenpeace 1991). Since the 1999 Kosovo War, and often led by the UN Environment Programme (UNEP), post-conflict environmental assessments (PCEAs) have sought to take a more holistic approach, documenting a wider range of direct and indirect forms of harm (Jensen and Lonergan 2012).

United Nations-led assessments, which can only take place upon the invitation of the affected state, help to address the politicisation of conflict-linked environmental harm by seeking to provide impartial information on the damage identifiable at the time of the assessment. However, in inviting an assessment, the motivations of the affected state may vary and can include a wish to seek reparations for damage or to attract financial support for recovery, as well as concern over the impact of the conflict on their population and environment. As assessments are financed by other governments, while the findings may be neutral, the process behind the assessment remains subject to politicisation.

Should an invitation be made, and where funding is available, comprehensive assessments also require safe access for UN experts and national partners. Security considerations therefore place a temporal barrier on field data collection. As the majority of contemporary non- or quasi-international conflicts have lasted for several years, or have been followed by prolonged periods of low-level conflict or insecurity, this can result in significant delays in assessment between the onset or conclusion of conflicts and sampling campaigns, resulting in gaps in data collection that may restrict analysis of changes in environmental quality over time. One solution to this problem that has previously been employed by UNEP is to provide capacity building for national environmental experts outside the affected country (UNEP 2007a). But for states with serious and widespread environmental problems, this may only be a stopgap measure. Another solution is for narrowly focused emergency assessments of high-risk sites, where security conditions allow; for example those increasingly undertaken by a partnership between UNEP and the UN Office for the Coordination of Humanitarian Affairs (OCHA) or UNEP’s 2017 rapid technical assessment of areas retaken from Islamic State in Iraq (UN Environment 2017).

This may however mean that assessments can be limited in their geographic scope, focusing on locations of highest perceived risk or political concern and, while findings are published and recommendations made to national authorities, governments are under no obligation to address the problems identified, and follow-up may be limited. Similarly, while assessments may highlight potential health risks, efforts by national authorities or relevant international organisations to follow-up these findings with research into health outcomes are rare.

The practical, political and financial limitations that the UN and other intergovernmental bodies face in assessing the environmental and derived humanitarian consequences of conflicts have implications for the protection of human health and ecosystems. They also constrain efforts to strengthen policies intended to minimise the environmentally harmful conduct of state and non-state militaries. While there are a growing number of entities interested in documenting these consequences, including intergovernmental organisations, national authorities, humanitarian and environmental NGOs, all face limitations in the collection and utilisation of field data. This has created dependence on remote data collection, and with it, a need for new methodologies that can bypass the systemic limitations faced by formal assessments (e.g. Witmer 2015; Levin et al. 2018).

While data collection during conflicts is vital to document incidents and to address the low priority afforded to environmental protection in times of war, it is also critical in the post-conflict phase. Typically, what media and governmental attention that can be built up for environmental concerns during conflicts rapidly diminishes afterwards. Communities may be left with serious health and environmental problems that weakened and distracted national authorities may be unwilling or unable to deal with (e.g. Kevany et al. 2012; Zwijnenburg 2013). National environmental authorities may have limited capacity even prior to conflicts, and which is degraded further during them. Environmental ministries may also be handed over to opposing factions as part of peace deals or their assistance measures may be used as a political tool and so, fail to reach all affected communities, increasing distrust in national authorities.

In addition to international organisations such as UNEP, OCHA and the Organisation for Security and Co-operation in Europe, some civil society organisations collect and publish environmental data during or after conflicts. A number of humanitarian organisations also collect data, but inter-organisational data sharing has historically been underdeveloped and uncoordinated (EHA CONNECT 2019). For humanitarian responders, those assessments that are undertaken primarily relate to the impact of their operations on the environments and communities that they are active in or the specific context of their activities within the humanitarian cluster system. Although efforts are underway to strengthen and coordinate environmental assessments in humanitarian response (Environmental Emergencies Centre 2017), it is clear that an important gap in field data collection exists, and that this gap is constraining efforts to document and address the health and environmental consequences of wartime environmental degradation and the concerns of the communities affected.

### Constraint 2: Baseline data for comparison

The absence of baseline environmental data against which to compare pre and post-conflict conditions is a perennial problem. At times this has been highly politicised, for example in the case of the NATO bombing of the Pancevo facility in Serbia (UNEP UNCHS 1999). The extent of the damage caused by the actions of a belligerent has implications for efforts to seek redress, as well as international assistance for remedial measures and the extent of any clean-up required. Although it remains context specific, many countries do have national monitoring systems in place for air or water quality, or for assessing the conditions around particular facilities. Nevertheless, many countries at risk from or affected by conflict do not, and some that do may be unwilling to make such data available to the international community due to domestic, commercial or wider political concerns; for example Russian refusal to confirm release of sulphur dioxide from a titanium plant in Crimea (Weir and Denisov 2019).

### Constraint 3: Complex environments and enigmatic impacts

The above considerations relate to the ability to collect data, though it should be noted that a further constraint is being able to confirm that the impacts are the result of armed conflict and to quantify them accordingly. Socioecological systems are complex (Whitfield et al. 2011) with multiple linkages and drivers between social and ecological components that interact across varied space and time scales. Establishing that impacts have been caused directly or indirectly by armed combat (or indeed any specific driver) can be problematic given these multiple interrelationships, and particularly for any hidden or ‘enigmatic impacts’ (Raiter et al. 2014) that may occur, and which fall ‘under the radar’ of more traditional environmental impact assessments and mitigations. These include (1) ‘cumulative’ impacts, which are negligible alone but become significant as they add up over space and time, such as repeated minor pollution incidents; (2) ‘offsite’ impacts, where impacts are removed from the site of interest and therefore fall out of scrutiny; (3) ‘cryptic’ impacts, where detection is just not possible given available technology, resources or knowledge and (4) ‘secondary’ impacts, which are those that are far removed from initial cause—an example would be road development leading to an increase in animal poaching rates (Barnes et al. 1995; Raiter et al. 2014).

Combined with the constraints given above, accounting for meaningful environmental harm from armed conflict becomes even more difficult, especially using traditional means of environmental assessment and by ‘outside’ agencies, yet is becoming increasingly important.

As Francis and Krishnamurthy (2014) have noted, new approaches to impact and quantification of impact are needed to satisfy international humanitarian law’s current provisions for environmental protection during conflicts, wherein ‘widespread’, ‘long-term’ and ‘severe’ impacts need to be demonstrated; terms which are themselves problematic and not well defined (UNEP 2009). Recent efforts to clarify and progressively develop the legal framework protecting the environment in relation to armed conflicts, which have also considered practices before and after conflicts, and the simultaneous applicability of human rights and environmental law, are making the question of damage quantification ever more important (ILC 2011).

It is increasingly recognised that broad environmental impact assessments, which tend to focus on a limited number of indicators, are unlikely to capture the full suite of environmental impacts, particularly in relation to the more enigmatic impacts and in an armed conflict situation (e.g. Lawrence et al. 2017). Several authors have drawn attention to the need to develop more integrated and participatory assessment approaches, incorporating a range of stakeholders and multiple sources of knowledge to ensure legitimacy of assessment (Whitfield et al. 2011; Barua et al. 2013). Certainly, the value of local ecological knowledge (Davis and Wagner 2003; Aswani et al. 2018) in determining impacts is likely to be invaluable and can only come from local stakeholders who are actively using the environment and are therefore best placed to identify the types of impacts that may have occurred and how this may have led to more cryptic, enigmatic influences on environment, health, livelihoods and quality of life (for instance).

There have arguably been two significant innovations that offer opportunities for overcoming the constraints discussed above. The first is remote sensing technologies, which have increased their capabilities significantly in recent years. The second is the utility of technologically mindful citizen science activities to detect and quantify local impacts on the ground. We explore these two innovations below, in the context of the impact of armed conflicts.

## Innovations for the collation of environmental information in armed conflict situations

As the political and environmental context of each conflict is unique, and the system of UN-led assessments is largely ad hoc and still under development, there is scope for innovation in environmental data collection during conflicts. This need is underlined by recent trends towards deliberate damage to environmentally risky infrastructure in conflicts in the Middle East and North Africa region (Sowers et al. 2017), and the fact that a number of environmentally harmful military practices, such as the targeting of oil facilities, remain commonplace (Bohm 2015).

### Remote sensing of conflict environments

Remote sensing is the measurement and/or collection of information about objects or spatial areas from a distance, and typically involves imagery collected from aerial photography or satellite technology. There has recently been an expansion in the use of remote sensing to identify armed conflict and warfare activities, sources and environmental impacts, in some cases in conjunction with modelling data (Gorsevski et al. 2012; Witmer 2015; Björnham et al. 2017). Witmer (2015) notes that this growth is partly the result of advances in remote sensing technology that can provide high-resolution (e.g. < 1 m) imagery with short revisit periods of (for example) 2–4 days, meaning that changes can be rapidly detected, along with an increase in the quality and availability of free online imagery, such as that utilised by Google Earth (imagery provided by Digital Globe) and Sentinel Hub (which uses Sentinel-2 data from the European Space Agency, and Landsat-8 and MODIS data from the National Aeronautics and Space Administration), as well as the emergence of significant, complex and protracted conflicts such as in Darfur in Sudan, in which the need for remote sensing assessments was apparent.

In his review, Witmer (2015) organises impacts from armed conflicts into temporal categories, indicating that environmental effects are observable at the scale of hours to days, in contrast to aspects like population movement (days to months) and land cover change (months to years). Environmental impacts, especially enigmatic impacts, may of course take months, years or even decades to fully materialise or be acknowledged. For example, Witmer (2015) notes the use of land and resource degradation for the detection of internally displaced people and refugee camps. Nonetheless, the hours-to-days timeframe is indicative of the main focus of remote sensing on conflict and the environment, which is highly visible pollution incidents resulting from the burning of oil wells and lakes, or forest fires (Witmer 2015).

This increased focus on remotely sensed data has partly been driven by necessity, such as for military health surveillance, and partly by the temporal constraints in traditional PCEAs. The shift has also coincided with the rapid growth in the use of open-source intelligence (OSINT) by NGOs and other non-state actors to monitor conflicts, in particular, human rights violations (Koettl 2017). Satellite analysis, coupled with OSINT data, has already demonstrated its potential in the early identification of environmental hazards in conflict settings. When coupled with generic hazard assessment tools, such as OCHA’s Flash Environmental Assessment Tool, it is possible to remotely compile indicative information on the locations of potential environmental risks, including in conflict contexts (UN HABITAT 2017; Zwijnenburg 2015). These locations can then be prioritised for later assessment and used to inform post-war recovery planning.

While these remote data collection methodologies will be an increasingly valuable tool for identifying some forms of environmental damage in conflict settings, particularly pollution hazards, they have their limitations. Information is restricted to what is visible over the timeframes of available sensors, and local knowledge on the location or disposition of damaged or hazardous sites may be necessary to validate findings. Alongside this, data necessary for accurately determining health and environmental risks are often generalised, rather than specific; and findings are still reliant on later field-based assessments. The politicisation of environmental information (i.e. presenting information in a way that supports a political agenda) during and after conflicts also poses a challenge to remote data collection (see Weir and Denisov 2019), with international organisations constrained in how they can utilise data that has not been verified by field analysis. Other sources of data, such as that collected by military actors deployed to conflict areas to inform forces health protection, are rarely made accessible to non-military parties (Garrity 2015).

One of the most dynamic aspects of the expansion in satellite imagery has been the growth in citizen participation. The field of crisis mapping (e.g. Shanley et al. 2014) allows volunteers in country and globally to take part in mapping vital infrastructure and transport routes after events like hurricanes or earthquakes, by translating satellite photos into operational maps (Kerle 2012; Humanitarian OpenStreetMap Team 2018). The enabling platform in this case is OpenStreetMap (OpenStreetMap Community 2018), the crowdsourced global mapping base layer. In a similar manner, projects like SkyTruth and PublicLab have used participatory methods based on satellite imagery to track environmental threats such as fracking (SkyTruth 2014) or the flooding of toxic Superfund sites after hurricanes Harvey & Irma (Public Lab 2017). A parallel use of crowdsourcing and satellite imagery can be seen in OSINT/open-source investigation projects such as Bellingcat (Bellingcat 2019). While this activity has been primarily focused on identifying the parties responsible for events such as the shooting down of airline MH17 (Bellingcat Investigation Team 2019) or chemical weapons attacks in Syria (Higgins 2017), there is also a strand that has a focus on environmental risks and damage (Bellingcat Investigation Team 2015; Khachatryan 2017; Zwijnenburg 2018).

While satellite imagery has opened up the possibility for participation and the development of citizen expertise, it lacks the direct connection to ground truth that is essential for accurate analysis of impacts. It is notable that the most successful open-source investigations have combined the analysis of satellite imagery with volunteers or activists on the ground who have been able to go to the locations of interest to collect visual and empirical evidence (Bellingcat Investigation Team 2018; Triebert 2017). While remote monitoring is an important part of the solution, it needs to be complemented by action on the ground.

### Citizen science and conflict: the emergence of civilian science

Participation of members of the public in scientific inquiry is not new, and there is a long history of amateur enthusiasts collecting observations of nature for scientific interpretation (Resnik et al. 2015; McKinley et al. 2017). However, citizen science is a rapidly expanding field that describes people’s involvement in a wide range of activities, from large-scale information gathering to social justice activism, enabled by the affordances of the Internet and low-cost sensing technologies (Ellwood et al. 2017). Professional citizen science societies have been established in the USA, Europe and Australia, and citizen science models are increasingly being incorporated into the activities of governmental and non-governmental agencies (Ellwood et al. 2017). The ethos of citizen science is openness, sharing and participation across the whole scientific process (Resnik et al. 2015). A good example of citizen science in a crisis context is the response to the Deepwater Horizon oil spill in 2010, where citizens developed low-cost do-it-yourself (DIY) technologies such as kite mapping to monitor and track the spread of oil pollution (McCormick 2012). The potential of citizen science and crowdsourcing data was recognised by the Obama administration in the USA, which developed a federal toolkit (Gustetic et al. 2014), and the US Environmental Protection Agency, which is ‘working with agency researchers to help bring local air measurement capabilities to communities’ (Kaufman 2015).

One of the underlying values of citizen science is the inclusion of local knowledge and local perspectives. This is both pragmatic and epistemological. Observations from people on the ground and immersed in a particular context can provide vital missing clues to any investigation, especially in post-conflict situations where access for professionals is limited. But beyond that, the more empowering forms of citizen science recognise the value of situated knowledge (Haraway 1988) and question the idea that the universalising ‘view from above’ of mainstream science can immediately capture nuances that are important to excluded social groups such as women or people of colour (Harding 1998). This postcolonial strand in citizen science may help to give it traction in post-conflict situations, where communities have experienced exclusion or are sceptical about the motives of various state actors (Garrity and Zwijnenburg 2016). The environmental justice movement in the USA has shown that communities of colour living in areas historically dominated by highly polluting industries (‘fenceline communities’) have been able to push back against a corporate and institutional narrative that there is no cause for alarm. The Clean Air Coalition in Tonawanda, New York, used citizen science to show that high levels of benzene were coming from a local foundry coke plant. This eventually resulted in EPA legal action and fines and prosecutions for the company (U.S. Environmental Protection Agency 2013).

Community citizen science (Chari et al. 2017) is a form of critical pedagogy, where people in the community are fully involved in asking the questions about their environment, carrying out the investigations and deciding how to act on the results. The agency this gives to participants has particular relevance in a post-conflict situation, when there is likely to be an absence of governance and capacity in terms of the resources or issues that are being addressed. The forms of self-organisation typically used by existing community citizen science projects are horizontal and democratic. This agency and self-organisation can also contribute to the general resilience of the local population. The citizen coalition that discovered the benzene release in New York State went on to develop community participatory budgeting to decide how the settlement money from the corporate fines would be spent on local projects (Mistretta 2013; McMurray 2018).

Despite the growing acceptance of citizen science, some parts of the scientific community object that science carried out by people with an advocacy agenda (i.e. people directly affected by an issue) will be prone to potential bias. This stems from the historical self-image of scientific culture as embodying neutrality and objectivity and tends to overlook the unconscious institutional or cultural commitments of professional scientists themselves. In any case, the remedy here is to focus on the process rather than the people; if the methods are demonstrably scientific and the analysis takes into account sampling issues, then the results can stand in their own right (e.g. Bonney et al. 2009; Haklay 2015).

The other frequent objection to citizen science is to the data quality, expressing a doubt that people without formal scientific training are able to collect data that is consistent enough to be meaningful. This is contradicted both by historical volunteer data collection and recent studies, which indicate that citizen science can produce reliable data, whether in specific studies (Palmer et al. 2017) or as a general field of data collection (Follett and Strezov 2015). The aim in a post-conflict situation is not to produce a peer-reviewed scientific paper but to be able to indicate with reasonable probability where there may be a significant problem that requires attention and intervention, in a similar way to investigations by ‘fenceline communities’ in the USA.

Whatever the specific aims of the citizen science project, the basic requirement is systematic observation or measurement. In contexts where the mode of observation is making notes or using a smartphone to take photos, consistency of data can be achieved through agreeing a protocol and by providing some means to verify the evidence. The Save Our Streams project in Pennsylvania used smartphone photos and GPS data to map lost and forgotten oil wells that leak methane, and which can also become a major hazard when disturbed by fracking operations (Moskowitz 2014). This is sometimes supplemented through the use of a handheld methane detector. In general, taking measurements is more challenging than observation and mapping because of the need for some kind of calibration. It is unlikely that post-conflict situations will provide the opportunity to test devices or sampling methods against local standards-compliant sensors, so the methods used must be robust enough to overcome this constraint and provide a chain of reference between their readings and some form of verified comparator.

However, it is also important that post-conflict citizen science is able to adapt to the notion of ‘standards’ to fit the particular context and need. Most standards for pollution or toxicity that have been adopted in regulatory frameworks reflect both peacetime settings and negotiation between government and industry. Even in non-conflict situations, this may obscure community concerns. A case in point are the laws on air quality in the USA and Europe, which are expressed in terms of monthly or annual averages and population-level epidemiology, whereas the concerns of residents living next to an oil refinery may focus on short-term peak concentrations and the impacts on individual’s health (Ottinger & Zurer 2011). It is likely that post-conflict citizen science will be addressing situations outside of the range of normal standards regimes.

Another important feature of community citizen science that is relevant to post-conflict situations is the orientation towards action. Projects that are addressing environmental pollution, for example, are interested not only in making measurements but in reducing harm. This could mean advocating for intervention by institutions to address the source of the problem, but also includes direct means of ameliorating the impacts. For example:

- School children in Camden, London monitored NO_2_ levels in order to map their lowest pollution walking routes to school (https://www.camden.gov.uk/ccm/cms-service/download/asset?asset_id=3124366).
- A project in China developed cheap indoor air purifiers by strapping replacement HEPA filters to domestic fans (https://smartairfilters.com/cn/en/about-us/).
- As PCB and mercury pollution from defunct industrial sites threatened to completely disrupt the Akwesasne Mohawk peoples’ traditional culture, by stopping them fishing in local rivers, the Saint Regis Mohawk Tribe (SRMT) Fish Advisory project developed a guide for which breeds and sizes of fish could safely be eaten in a limited number of monthly portions (https://www.srmt-nsn.gov/_uploads/site_files/FishAdvisory_WebFinal.pdf).
- The Parent-Teacher Association at El Marino Language School in California, which is located within 500 ft. of the 405 freeway, raised funds to install air filtration systems in classrooms (https://elmarino.ccusd.org/apps/pages/cleanair).
- In Sunset Park, New York, youth members of UPROSE, Brooklyn’s oldest Latino community-based organisation, gathered air quality samples and tracked vehicle activity in areas associated with sanitation traffic. The activities, carried out in 2016, informed the campaign to transform the commercial waste industry in New York City (https://www.uprose.org/climate-justice/).

## Data sharing and utilisation

Although international interest in the environmental dimensions of conflicts has expanded significantly in recent years, with a number of parallel political and legal initiatives under development on a range of environmental security themes, the question of how knowledge and data can best be operationalised for the benefit of human health and ecosystems remains. The environment is still under-prioritised in international responses to conflicts and humanitarian crises, a matter being addressed with initiatives to enhance the use of climate and water-risk data in the early warning of conflicts (Conca et al. 2017), in measures to better integrate the environment in humanitarian response and in work to ensure that equitable and sustainable natural resource management policies are implemented post-conflict.

In light of the practical constraints on data collection inherent in conflict and post-conflict settings, and the methodological limitations of low-cost analytical technologies when compared to those deployable by states or international organisations, it is clear that the aim of civilian science should be to complement, rather than replace, more comprehensive assessments. In this, the objective should be the collection and dissemination of ‘just good enough data’ (Gabrys et al. 2016), which helps catalyse formal systems of assistance by providing indicative data on environmental harm and associated public health risks.

Assuming that it can be collected, methods for the verification, presentation and communication of environmental data must also be considered. For ‘civilian science’ projects to make a contribution to environmental protection and the alleviation of human suffering, mechanisms must be identified not only for presenting data but that also for ensuring that it reaches the relevant actors that can utilise it. In the context of armed conflicts, this would require the utilisation of existing response and development data frameworks.

Humanitarian agencies have been relatively quick to adopt big data platforms to track the impacts of crises to inform responses. For example OCHA’s open platform for sharing data, the Humanitarian Data Exchange, which launched in July 2014, utilises data from around 1000 organisations and entities and includes data relevant to the environment. Initially developed to provide an overview of natural resource management in fragile and conflict-affected states, the UN Environment, World Bank and Global Resource Information Database ‘Map X’ platform is increasingly being used to document a range of environmental themes (Lacroix et al. 2019). The platform offers users a variety of tools to map and present data, for both research and advocacy.

With free to use data and communication platforms now available, data collected by civilian science projects have the potential to be widely available to relevant agencies and actors, and in readily accessible formats. For data collected during or shortly after conflicts, analyses could be made available directly to specific humanitarian actors working on relevant issues with the humanitarian cluster system, such as water sanitation and hygiene, shared with a wider range of entities through multi-sector needs assessment mechanisms or through geographically focused response and recovery platforms such as those managed by UN-Habitat (UNHABITAT 2015).

However, while many humanitarian and development actors are engaged in the collection and utilisation of data relevant to their activities, data collection on environmental risks is less developed, as are the means to share environmental data between organisations, although this is an area currently being addressed (Environmental Emergencies Centre 2017). Civilian science, along with remote open source techniques, could contribute to this process, particularly, where community level projects are undertaken in partnership with humanitarian and development actors.

To ensure the acceptance of data collected in areas subject to political contestation, consideration will be needed both in the design of projects and in the dissemination of data. At the project level, this should involve the utilisation of methodologies with a high degree of acceptance in peacetime citizen science, or which are used or endorsed by national regulators. Platforms for the public dissemination of data should include mechanisms to review findings and to ensure the security of participants. It is important that communities have some control over the data they have created. Dissemination should aspire to full transparency, although there may be circumstances where disclosure might be restricted if it is in the best interests of the participating community or in facilitating environmental or health assistance. Methodologies and their limitations should be clearly described alongside the results.

Given the often lengthy delays in the provision of post-conflict assistance, be it formal assessments or environmental remediation programmes, projects should provide for short as well as long-term measures that utilise data to reduce health and environmental risks. For example, building a risk awareness component into projects could help to inform short-term behavioural changes among affected communities, in turn reducing the risk of exposure to particular toxic remnants of war.

A further consideration in the design of projects is their utilisation for environmental cooperation between affected communities. Where environmental risks or damage extend over a wide area, which may encompass front lines or contested areas, community concerns may be shared. This raises the possibility of citizen science projects being used as a tool for environmental peacebuilding to prevent harm to communities or damage to shared resources, such as aquifers. This can build on the perception of the environment as a comparatively unpolitical object of common concern and an entry point for discourse in contested settings (Yaari et al. 2015).

## Potential applications for civilian science damage monitoring

The specific footprint of environmental damage associated with a given conflict varies widely and is typically unique to each conflict. Historically, short-term, high intensity international armed conflicts have often seen the generation of serious pollution threats, while long-term, low-intensity civil wars have been associated with natural resource degradation and the indirect environmental consequences of the collapse of governance (ILPI 2014). However, the hybridised conflicts of the last decade have often blurred this distinction.

Nevertheless, there are common forms of harm that occur regularly during conflicts, irrespective of their nature, and which are often associated with both acute and chronic impacts on communities. Three of these are land degradation, the environmental legacy of explosive weapons’ use in urban areas and pollution linked to oil infrastructure. Potential methodologies for the participatory monitoring of these problems are discussed below. All three are generally identifiable by remote sensing but they are also forms of damage where ground-based observations would make a significant contribution to understanding their livelihood, health and ecological risks, and in informing health or environmental interventions.

Any discussion on potential methodologies must also consider the means through which participants would benefit from the project, both in the short and longer terms, and the practical barriers presented by access and security issues. Ethical issues are important; for example, the act of data collection, such as that related to potential toxicity and subsequent health impacts, could result in increased fear and anxiety if the context for the collection is not properly communicated or if the policies or protocols for follow-up actions by relevant organisations or authorities are unclear (e.g. Cullen 2011). Practical barriers include, for example, the ability to transport equipment into and out of affected areas and the safety of the participants. Equipment considerations should include ease of use, robustness, reliability, cost and the ability to function without mains electricity. This will inevitably necessitate that a balance be struck between the accuracy of sensors and their utility in these settings.

Although the security conditions of many conflict and post-conflict settings present considerable challenges, the increasing use of citizen science or community-led approaches for monitoring environmental hazards in insecure or politically contested contexts (Phys.org 2018), or simply to bear witness to harm (Fiske 2018), suggests that these challenges may not be insurmountable.

Similarly, while the environment may be a low priority for many living in areas affected by armed conflict, there is an increasing number of examples of where individuals, local civil society or community groups have continued to implement environmental projects during conflicts or contributed to humanitarian initiatives to map environmental resources or to assess damage (ICRC 2015b). Nevertheless, it seems likely that an external entity may have to play an enabling role, be it through training, the provision of equipment or capacity building. Support and facilitation for community-level participatory projects could be provided by local civil society organisations, first responders such as mine action and humanitarian NGOs, academic institutions and relevant international organisations (UNEP 2018).

### Monitoring land degradation

Land degradation is a common impact from armed conflict and military activities in general and can result from (1) explosive ordnance or the use of military vehicles (Certini et al. 2013), which can damage or remove vegetation cover and destroy soils; (2) contamination of soils with pollutants such as oils, explosive residues and heavy metals (e.g. Meerschman et al. 2011) and (3) the displacement or intensification of resource-based land use practices such as crop production, grazing or timber extraction, which in turn can lead to longer term declines in ecological health and soil quality, with some soils in particular taking decades to centuries to fully recover, if they can at all (Certini et al. 2013; Sánchez-Cuervo and Aide 2013; Ingalls and Mansfield 2017). Such declines can compromise the socioecological resilience of an ecosystem and exacerbate future problems and conflict, as capacity and potential for resource production are reduced, or by necessity focused elsewhere (Ayana et al. 2016; Ingalls and Mansfield 2017; cf Theisen 2008).

Physical (geomorphological) impacts to land surfaces, vegetation and soils may be observed from satellite imagery and as necessary confirmed with simple ground truthing, at least establishing the area and extent of the impact, even decades after the conflict responsible (e.g. Hesse 2014). Chemical contamination of soils is harder to determine and relies more on a mix of in situ measurements (which can be performed for simple indicators such as nutrients and sometimes VOCs) or laboratory analysis for more complex chemicals such as heavy metals, PAHs and pesticides. Water contamination may be detectable from satellite imagery (Gholizadeh et al. 2016) but field samples are often required for soils and need to be of sufficient number and size for an accurate understanding of impact to be determined (Davidson and Williams 2009).

Likewise, the identification of changes to soil structure, fertility or biodiversity also relies on field measurements, though these can be relatively simple, such as visual inspection of soil types, horizons, organics, particle size and indicators such as earthworm species and abundance and can be linked to digital soil mapping (Rossiter et al. 2015). In particular, there is potential for photo imagery or dedicated apps, as provided by smartphone technology, to be used to visually assess and record amount and type of degradation, from damaged and eroded soils and changes in vegetation cover, to evidence of pollution such as oil-soaked soil (Roy et al. 2012; Dehnen-Schmutz et al. 2016). Though this is still in its infancy, the potential exists to collate a multi-source evidence base to address conflict-related land degradation, which may be especially useful in hard to reach or contested areas.

There is notable value in local ecological knowledge for measuring, mapping and understanding land and soil degradation, particularly as local communities will understand the history and cultural values of land and soil use. Involvement of local stakeholders is much more likely to result in the identification of key affected areas, and both ecological and cultural impacts. Sharing of knowledge, expertise and data may help prevent displacement and encourage the right type of post-conflict mitigation and restoration in the right areas, in combination with NGOs who may be aiming to help with post-conflict rehabilitation (Milburn 2012). The self-organisation of local people to both measure the problems and advocate for action at local and national levels can be part of reconstitution or even flourishing of post-conflict civic culture.

### Monitoring the legacy of explosive weapons in urban areas

The use of explosive ordnance in urban areas, with often indiscriminate wide-area effects, is common to many conflicts. Alongside the direct civilian harm these practices cause, the intensive use of explosive force results in both direct and reverberating damage to essential environmental services such as water and sewerage networks (ICRC 2015a), damage to light industrial and residential properties, and the generation of large volumes of debris, which may be contaminated with asbestos, furans, heavy metals, explosive residues and be of a readily respirable size (UNEP 2007b; TRW Project 2014).

In spite of the near-ubiquitous nature of this form of conflict pollution, data on the characteristics and composition of the pulverised building materials created by the use of explosive weapons are largely absent from the literature. Collecting data on its properties and exposure risks, and documenting the general environmental legacy of the use of explosive weapons in urban areas, such as the locations or damage status of environmentally hazardous sites, would be of considerable value for informing risk assessments and determining early recovery priorities. Data collection could be undertaken during conflict, in its immediate aftermath and continue into the recovery phase.

Community-led mapping of damage to buildings, industrial areas and debris could make use of pre-existing tools with limited modification. For example, the ICRC’s platform for mapping functioning water systems that were used in Aleppo (ICRC 2015b) or the UNDP’s Redonbass platform and app that was developed to map damage to buildings and infrastructure in eastern Ukraine (UNDP 2015). Redonbass created an online platform where users could upload geotagged photos of damage via a smartphone app as a means of documenting the intensity of destruction and so facilitating repairs and clearance. A platform of this type could be readily repurposed to include damage to water and sanitation infrastructure, areas affected by debris or damage to industrial sites in urban areas. However ensuring that risks to participants were managed would be critical in light of the potential presence of explosive or toxic remnants of war.

Analysis of the composition of pulverised building materials, whether for combustion products, asbestos or heavy metals currently requires access to an analytical laboratory. While broad-spectrum air samplers could be appropriate for conflict contexts (e.g. AQMesh 2018) and provide indicative data on PM_10_ and PM_2.5_ levels—as well as a range of gases—they would need to be complemented by methodologies for sample collection and analysis. One method that has been used for wide-area urban air sampling has been the collection and analysis of vehicle air filters (Rivera and Rodriguez 2016). The used filters, which could be sourced from the vehicles of humanitarian or demining organisations, are readily transportable and their contents can be subject to chemical and optical analysis in a laboratory. However, for civilian citizen science to be empowering, it is important that participants are not just involved in collecting samples but in inclusive discussion about what the analysis shows and what the priorities for action should be.

### Monitoring oil pollution

Recent and ongoing conflicts in the Middle East and North Africa have seen widespread damage to oil infrastructure, most notably in Iraq where Islamic State used oil facilities as a weapon of war. The Iraqi town of Qayyarah was particularly heavily affected by oil well fires between 2016 and 2017 (UN Environment 2017), exposing both its residents and IDPs fleeing Mosul to pollutants for months. Significant health concerns were particularly related to the acute risk of exposure to sulphur dioxide from the burning Mishraq facility, which killed a dozen people and affected more than one thousand (Joint UNEP/OCHA Environmental Unit et al. 2016). No environmental sampling was undertaken during the fires, in spite of OCHA suggesting that portable air monitoring equipment be installed by the Iraqi government (Joint UNEP/OCHA Environmental Unit et al. 2016; TRW Project 2016). Similarly, in both Iraq and Syria, there are also significant concerns over the growth in artisanal oil refining, its health impact on workers and local communities and its long-term environmental impact (Zwijnenburg 2016). The severity of oil pollution in these conflicts, and the technical and financial burden it has placed on national authorities, means that remediation efforts will likely take decades. This will place affected communities at risk from long-term exposure to oil pollution from spills, soil and groundwater contamination.

Whether sampling during conflict or in its aftermath, passive diffusion tubes offer significant and well-established potential for monitoring a range of volatile organic compounds (VOCs) from oil pollution, whether from fires or spills (e.g. Gradko 2018). Cheap, simple to use and with the potential for determining atmospheric pollutant levels across a large geographic area and for up to a month, diffusion tubes have been widely used in citizen science projects monitoring air pollution. They do however require access to a laboratory for analysis and have a shelf life of 10 weeks, including the period of use. For monitoring emissions from oil fires or artisanal refining practices, the tubes could be teamed with broad-spectrum air samplers capable of measuring gases and particulate matter. Many citizen science air quality projects have demonstrated that these methods can be used as part of projects that mobilise active community involvement and engagement.

## Conclusion

In considering the potential role of participatory citizen science in the context of conflict and post-conflict settings, this paper has identified a number of means through which ‘civilian science’ could serve to enhance the environmental security of conflict-affected communities.

Foremost among these is its potential to help address existing limitations in field data collection methodologies in insecure or contested environments. By bypassing current formal systems of assessment, citizen science methodologies could reduce delays in data collection or contribute to data collection over longer periods and wider geographic areas than traditional assessments. Yet as they could also help to create the political space for formal assessments by international organisations, their role should be viewed as complementary.

The need to address limitations in data collection in conflict contexts has been a key driver in the recent growth of remote sensing, whether for monitoring human rights abuses or environmental harm. Citizen science methodologies hold great promise in complementing these approaches with ground truthing informed by local knowledge and experience. During conflict and early recovery, mechanisms to utilise data gathered by citizen science projects already exist within the humanitarian response system and are in the process of being strengthened to allow more effective sharing and utilisation of environmental data.

The collapse of institutions and governance during conflict has long-term consequences for environmental protection and the realisation of environmental human rights. The ability of participatory citizen science to provide affected communities with political agency, while at the same time, supplementing or filling gaps in the delivery of local environmental health services that the authorities may be unable, or unwilling to provide, is a compelling reason to explore further work in this field. So too is the question of its potential contribution to informing ongoing efforts to enhance the legal framework protecting the environment in relation to armed conflicts and to increase the accountability of both aggressor and affected states for the environmental damage caused by conflict, and for any subsequent measures to address it. Furthermore, the potential offered by the use of citizen science for raising community awareness of conflict-linked environmental problems and risks, and as a tool for environmental cooperation, could make it a valuable tool for civilian protection and peacebuilding.

In briefly examining three common forms of environmental damage linked to armed conflict, each of which has a demonstrable impact on human health or livelihoods, we find that the tools necessary for citizen science approaches are readily available, be they sensors or data collection and communication platforms. Where possible, context-appropriate methodologies should be developed in conjunction with the organisations capable of facilitating and implementing projects, to ensure that they can be easily accepted as part of their field activities.
